# A Multimodal Feature Fusion Brain Fatigue Recognition System Based on Bayes-gcForest

**DOI:** 10.3390/s24092910

**Published:** 2024-05-02

**Authors:** You Zhou, Pukun Chen, Yifan Fan, Yin Wu

**Affiliations:** 1 College of Information Science and Technology, Nanjing Forestry University, Nanjing 210037, Chinayffannjfu@163.com (Y.F.); 2 Shanghai Shentian Industrial Co., Ltd., Shanghai 200090, China; 3 Shanghai Radio Equipment Research Institute, Shanghai 201109, China

**Keywords:** EEG, ECG, feature fusion, Bayes-gcForest, fatigue recognition

## Abstract

Modern society increasingly recognizes brain fatigue as a critical factor affecting human health and productivity. This study introduces a novel, portable, cost-effective, and user-friendly system for real-time collection, monitoring, and analysis of physiological signals aimed at enhancing the precision and efficiency of brain fatigue recognition and broadening its application scope. Utilizing raw physiological data, this study constructed a compact dataset that incorporated EEG and ECG data from 20 subjects to index fatigue characteristics. By employing a Bayesian-optimized multi-granularity cascade forest (Bayes-gcForest) for fatigue state recognition, this study achieved recognition rates of 95.71% and 96.13% on the DROZY public dataset and constructed dataset, respectively. These results highlight the effectiveness of the multi-modal feature fusion model in brain fatigue recognition, providing a viable solution for cost-effective and efficient fatigue monitoring. Furthermore, this approach offers theoretical support for designing rest systems for researchers.

## 1. Introduction

As a common physiological state, fatigue is defined as a state of decreased physical or mental activity [1]. Traditional methods for detecting fatigue have predominantly relied on subjective reports and physical indicators. Since changes in physiological signals are objective, measurable, and specific for data signals, and their changes are not affected by human subjective will [2], detecting brain fatigue through changes in physiological signals has gradually become a mainstream objective assessment method in recent years [3]. Detecting the brain fatigue state by analyzing changes in physiological signals constitutes a quintessential pattern recognition problem [4]. In studies related to brain fatigue detection, commonly used physiological signals include the electroencephalogram (EEG), electrocardiogram (ECG), electromyogram (EMG), and electrooculogram (EOG) [5]. Among these signals, the EEG is regarded as one of the most reliable physiological signals currently applied to detecting the state of brain fatigue [6]. Meanwhile, alterations in heart rate variability (HRV) as measured by an ECG have also been proven to be related to the state of brain fatigue [7]. Consequently, machine learning methods have been primarily employed for conducting related research both domestically and internationally in recent years. For instance, Wang et al. [8] attained an accuracy of 88.85% in classifying cognitive fatigue states by extracting the power spectral density (PSD) features of EEG signals. Butkevičiūtė et al. [9] used ECG signals to detect the brain’s fatigue state, which achieved 94.5% accuracy. Mu et al. [10] achieved 94% accuracy in fatigue prediction by designing a novel gating feature fusion method to adaptively extract and integrate the ECG and heart rate variability (HRV) features. With the advancement of deep learning, the capacity for generalization and learning exhibited by deep neural networks (DNNs) is being progressively explored and integrated into the realm of pattern recognition. Sheykhivand et al. [11] used a combination of compressed sensing (CS) theory and a DNN based on EEG signals to achieve fatigue binary classification with an average accuracy of 94.2%. Based on ECG signals after a continuous wavelet transform (CWT), Rachamalla et al. [12] converted them into a scalar map image, which was fed as the input to a pretrained DNN model and achieved up to 88.31% recognition accuracy.

Previous studies have indicated that conventional machine learning techniques often grapple with inadequate generalization performance, complicating the task of managing the intricate brainwave alterations associated with brain fatigue. While deep learning offers superior generalization capabilities, its “black box” nature obscures the understanding of its operational logic [13], and the necessity to tune numerous hyperparameters significantly affects model efficacy. Moreover, deep learning models typically necessitate extensive datasets for training, yet there is a scarcity of comprehensive publicly accessible brain fatigue datasets [14], thereby restricting practical model performance. Consequently, devising algorithmic models that can adapt to small sample datasets has emerged as a pivotal research direction in the field.

Thus, as an alternative to deep learning, the multi-granularity cascade forest (gcForest), a model renowned for its excellent generalization on small sample datasets, was proposed [15]. It is based on the principle of bringing together multiple random forests of different types to make a joint decision on classification by majority voting, and this integrated approach substantially improves the generalization ability of the model. Currently, gcForest has been applied to some brain fatigue recognition tasks based on physiological signals. For instance, Fang et al. [16] computed the multi-class features of EEG signals and used gcForest as a classifier for emotion recognition, which improved the recognition accuracy by up to 19.53% compared with the traditional machine learning methods. Yan et al. [17] proposed an EEG rhythmic feature-based optimized deep forest emotion awareness recognition method, using the gcForest classification model for emotion recognition, which achieved 96.711% classification accuracy on the DEAP dataset, and also pointed out that the classification accuracy of multi-feature fusion is significantly higher than that of the single-feature method.

However, the gcForest model also presents limitations. Especially when processing medium-to-large-scale samples, the resource consumption for training and prediction escalates markedly as the number of cascade forest layers increases [18]. In addition, the model overly relies on manual experience in the hyperparameter optimization process, which increases the complexity of the operation and results in a large workload. Together, these factors constrain the application efficiency and generalizability of the gcForest model in certain scenarios [19]. Therefore, achieving a balance between prediction performance and the training of cascade forest samples is crucial for realizing an automated hyperparameter optimization approach. This balance is vital for enhancing model performance and conserving resources.

Building upon the foundational framework of the Bayesian optimization algorithm, specifically sequential model-based optimization (SMBO) [20], this study suggests integrating Bayesian optimization within the gcForest model for automated hyperparameter tuning. This integration aims to strike an optimal balance between prediction accuracy and computational efficiency, thereby enhancing model performance and robustness, minimizing the uncertainties associated with manual adjustments, and alleviating the workload.

## 2. Materials and Methods

### 2.1. System Architecture Framework

**Figure 1:**
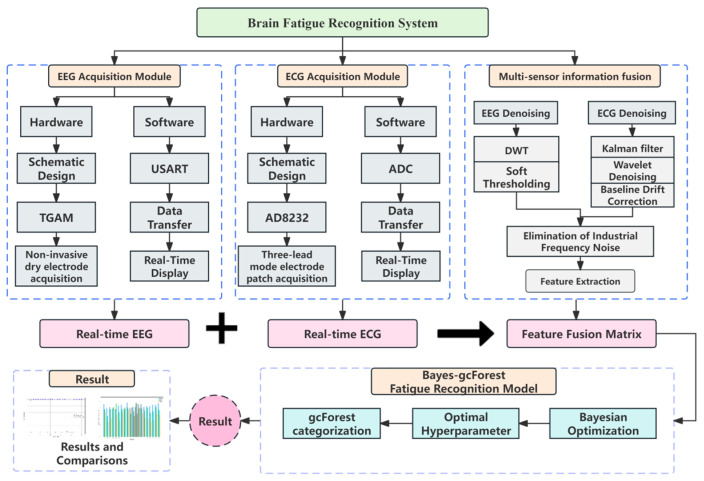
Schematic diagram of the basic architecture of a brain fatigue recognition system.

This study has developed an extensive system that includes embedded acquisition hardware, sophisticated host computer software, and carefully designed experiments for capturing raw signals. Through a series of steps encompassing data preprocessing and feature extraction, data derived from multimodal feature fusion are input into a refined gcForest algorithm-based fatigue recognition model. The application phase utilizes experimental data to validate the model’s effectiveness. The schematic diagram of the basic architecture of the system is depicted in Figure 1.

### 2.2. Acquisition System Design

#### 2.2.1. Hardware Module Design

**Figure 2:**
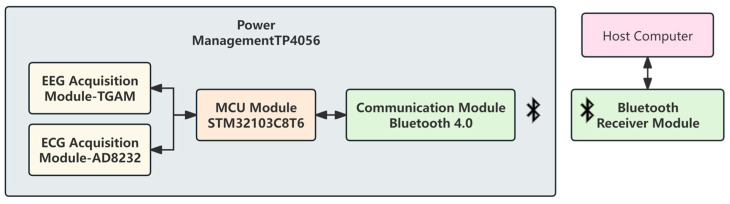
Hardware module flowchart.

The main components were a power supply system, a signal acquisition module, a micro-control unit (MCU), and a communication module. The design of the system’s hardware aims to achieve a balance between accuracy and reliability of data acquisition, while also prioritizing low power consumption and system stability. Efforts were made to enhance the portability and user-friendliness of the equipment. The hardware module flowchart is detailed in Figure 2.

Given that the system’s design necessitates a balance among portability, comfort, immunity to interference, and extended monitoring, its primary function is to evaluate cerebral fatigue states rather than conduct detailed clinical disease diagnoses. Consequently, the three-lead electrode patch method [21] was selected for ECG signal acquisition in this research. The ECG data obtained through this approach adequately provide for the analysis of fatigue-related physiological markers, such as heart rate variability (HRV).

**Figure 3:**
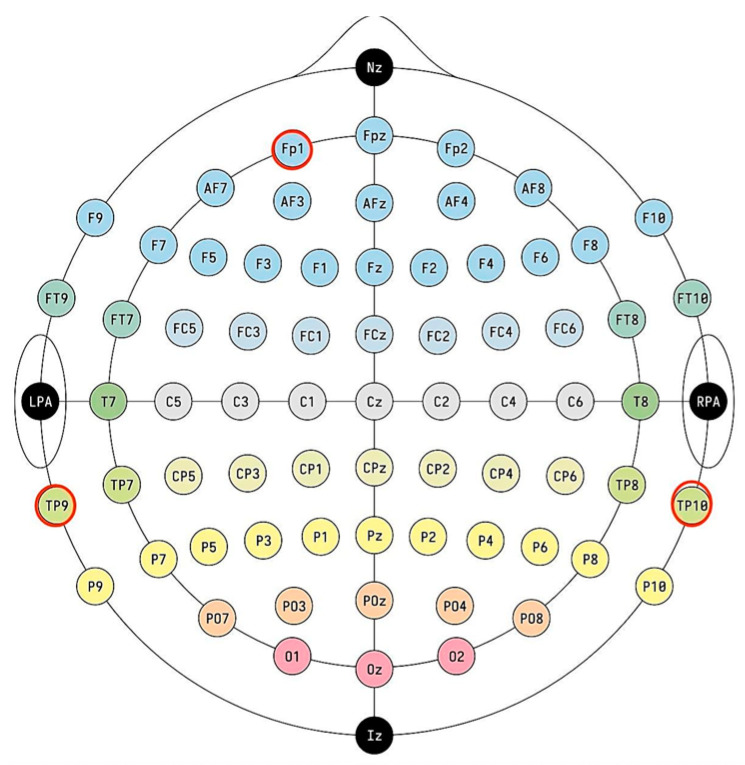
International 10/20 standard electrode positioning.

Some studies have shown that selecting a specific single-channel EEG can achieve up to 96.6% recognition accuracy, indicating the effectiveness of single-channel EEG in fatigue recognition [22]. Therefore, in order to facilitate the operation and reduce the cost of development, this paper uses a noninvasive dry electrode acquisition method that is more suitable for daily monitoring and portable devices. Referring to the International Electroencephalogram Society’s calibrated 10/20 electrode lead positioning standard, the EEG acquisition electrode was placed at a specific sampling point (Fp1) at the left forehead, which is usually not covered by hair [23], and the left ear (TP9) and right mastoid (TP10) were used for the reference electrodes, as shown in Figure 3. Then, the differential signals from the EEG electrode and the reference electrode were transmitted to the TGAM chip for signal processing.

**Figure 4:**
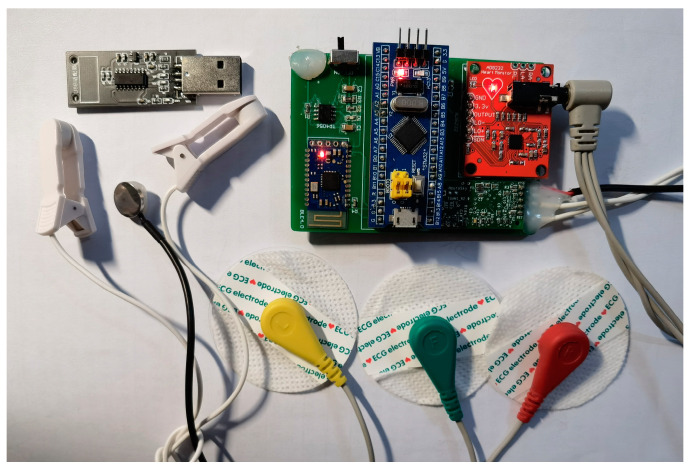
Photograph of hardware system components.

The system power supply module is tasked with the system’s power supply needs. It interfaces with the acquisition module which, upon capturing human body signals, utilizes the MCU for initial data preprocessing. The data are then transmitted to the host computer via the communication transceiver module for further processing. The hardware system’s design, as physically depicted in Figure 4, emphasizes a small, compact, and portable form factor.

#### 2.2.2. Host Computer

**Figure 5:**
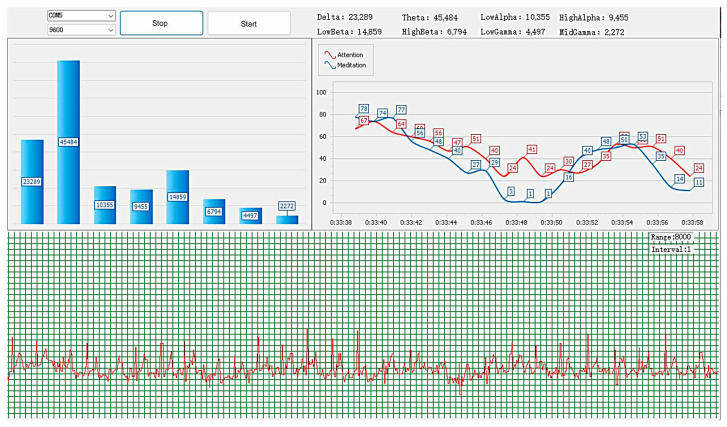
Signal’s real-time display interface.

EEG and ECG data collected by the system’s hardware are conveyed to the Bluetooth communication module through serial communication. The acquired data are then instantly relayed to the WinForms-developed software (version 1.0). The software’s interface, as depicted in Figure 5, facilitates the real-time visualization of various parameters, such as the wave amplitudes for the EEG and ECG signals across crossover bands. Additionally, this set-up permits instant storage of the data, which aids in the creation of a sample dataset pivotal for evaluating cerebral fatigue.

### 2.3. Data Acquisition Experiment

#### 2.3.1. Experimental Environment

The experimental configuration is shown in Table 1.

**Figure 6:**
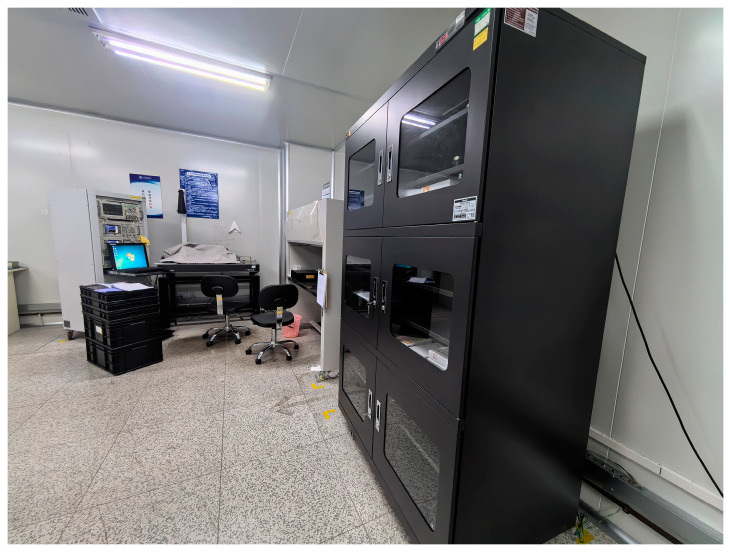
Experimental site.

To ensure high-quality signal acquisition during experiments, the selected venue was the electromagnetic shielding room at the Shanghai Radio Equipment Research Institute, as shown in Figure 6. This room is fortified with electromagnetic shielding material, providing an isolation barrier complete with a grounding line for robust protection against external electrical fields, magnetic disturbances, and electrostatic interference. Furthermore, the facility boasts an integrated constant-temperature air conditioning system, securing a consistent experimental temperature of 26 °C.

#### 2.3.2. Experimental Subject

This study involved 20 participants—university graduate students—balanced evenly with 10 males and 10 females aged between 22 and 25 years and distributed in different professional disciplines. All participants were thoroughly briefed on the experiment’s goals and protocols before consenting to partake. The specific selection criteria for the subjects are outlined in Table 2.

#### 2.3.3. Experimental Procedure

**Figure 7:**
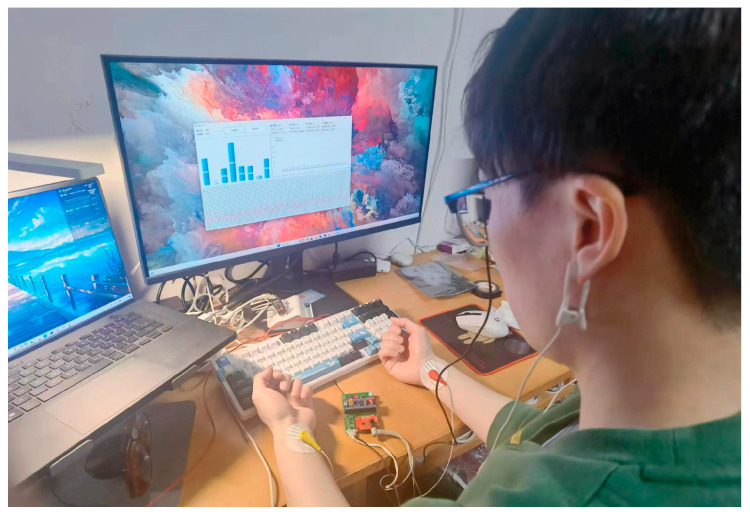
Experimental scenario.

Each participant was placed in a consistent indoor setting from 9:00 a.m. to 9:00 p.m. on weekdays, engaging in regular scientific research tasks or study activities. Physiological signal data acquisition occurred hourly, as depicted in Figure 7. This process involved collecting EEG and ECG signals at a sampling rate of 250 Hz, with each acquisition session lasting 5 min. In total, data from 20 individuals were gathered, resulting in 260 datasets of EEG and ECG signals. The collected data underwent preprocessing before being fed into the model for fatigue detection analysis.

Immediately after each data collection session, the subjects completed a subjective fatigue assessment scale. This study’s brain fatigue self-assessment tool was based on the Karolinska Sleepiness Scale (KSS), an established metric for assessing sleepiness across different conditions [24]. The scale ranges from 1 to 9, with a score of 1 indicating that the subject is in a state of high energy, vitality, and wakefulness. On the contrary, a score of 9 indicates that the subject is in a state of extremely serious drowsiness and high fatigue.

Referring to the European Union’s “Technical Requirements for the Driver Fatigue and Attention Warning System DDAW, CDR(EU) 2021/1341, 23 April 2021”, “With regard to the fatigue level of 6 or higher, it can be recognized that the participant has a degree of drowsiness, and the system should be accessed to the warning” [25]. All samples were labeled as “fatigued (Y)” and “not fatigued (N)” as shown in Table 3.

### 2.4. Signal Preprocessing

Owing to possible disruptions during the initial and concluding 30 s of signal acquisition, analysis was confined to the stable signals captured in the central 4 min of each experiment. These samples were categorized as “fatigued” (labeled as “Y”) and “non-fatigued” (labeled as “N”) for the purpose of this study. The protocol for signal preprocessing is detailed as follows.

#### 2.4.1. ECG Signal Processing

The original ECG signal $V_{ECG}$ was initially subjected to Kalman filtering to remove random noise, followed by a discrete wavelet transform (DWT), where the signals $V_{ECG}^{\prime }$ underwent multi-level wavelet decomposition. Noise components were eliminated based on predefined thresholds, and the signal was then reconstructed. After denoising, the signals were further processed for baseline drift correction to eliminate low-frequency drifts caused by respiration or limb movements, and the denoised ECG signal $V_{ECG}^{\prime \prime }$ was obtained as shown in Equations (1) and (2):

(1) $$V_{ECG}^{\prime }=KalmanFilter(V_{ECG})$$

(2) $$V_{ECG}^{\prime \prime }=BaselineCorrect(DWT(V_{ECG}^{\prime },\Psi (t),\varphi (t)))$$

(1) Kalman Filter

This process can be divided into the following two steps: prediction and updating, where the prediction process is formulated as follows in Equations (3) and (4):

(3) $${\hat{x}}_{k}^{-}=A{\hat{x}}_{k-1}+Bu_{k-1}$$

(4) $$P_{k}^{-}=AP_{k-1}A^{T}+Q$$

where ${\hat{x}}_{k}^{-}$ is the state estimate, A is the state transfer matrix, B is the matrix that transforms the inputs into states, $u_{k-1}$ represents the measurement noise, $P_{k}^{-}$ represents the covariance matrix prediction, and Q is the covariance. The updated formula is shown in Equations (5)–(7):

(5) $$g_{k}=\frac{P_{k}^{-}h^{T}}{hP_{k}^{-}h^{T}+R}$$

(6) $${\hat{x}}_{k}={\hat{x}}_{k}^{-}+g_{k}(Z_{k}-h{\hat{x}}_{k}^{-})$$

(7) $$P_{k}=(1-g_{k}h)P_{k}^{-}$$

where $g_{k}$ is the Kalman gain coefficient, h is the scaling factor, $Z_{k}$ is the actual measurement of the ECG, and R is the average of the measurement noise of $u_{k-1}$.

(2) Wavelet Denoising

Wavelet denoising employs wavelet transforms to refine signals by converting them from the time domain to the wavelet domain, where denoising occurs, followed by a reconstitution into the time domain. This method yields a time–frequency representation, ideal for analyzing non-stationary signals, and is adept at enhancing signal quality by mitigating interference [26].

The first step is to select the appropriate wavelet function. In order to enhance the ability to capture signal details and maximize the retention of important physiological information, this paper uses the Daubechies series wavelet, which is more suitable for signal feature extraction, as the mother wavelet and sets the decomposition level to 4 layers [27]. The advantages of Daubechies wavelets in denoising and feature extraction include the following:

(1) Multi-scale analysis: They are capable of capturing the details and overview of a signal at multiple scales simultaneously, which is suitable for the analysis of complex signals.

(2) Energy compression: Daubechies wavelets are able to concentrate the energy of the signal in a few coefficients, making the features more prominent for subsequent processing.

(3) Edge preservation: This type of wavelet is better able to preserve these features when processing signals with sharp jumps or edges, which is especially important for biomedical signals.

The wavelets of the Daubechies series are defined by the number of their vanishing moments. The more vanishing moments there are, the higher the smoothness and resolution of the wavelet. In specific applications, db4 is a popular choice because it provides well-balanced performance for a wide range of physiological signals. The wavelet decomposition is shown in Equation (8):

(8) $$x\left[t\right]=\sum_{k}cA\left[k\right]\cdot \emptyset_{J,k}\left(t\right)+\sum_{J=1}^{J}\sum_{k}cD[j,k]\cdot \psi_{j,k}(t)$$

where $x\left[t\right]$ is the signal that has been processed by Kalman filtering, $cA\left[k\right]$ is the approximation coefficient of the Jth layer, cD[j,k] is the detail coefficient of the jth layer, and ∅ and ψ are the scale function and wavelet function of the wavelet, respectively.

When performing the wavelet transform, it is necessary to set the appropriate number of decomposition layers J. This choice depends on the characteristics of the signal and the desired resolution and noise level. In general, the more layers of approximation coefficients there are, the larger the time scale of the analysis which is suitable for capturing the low-frequency characteristics of the signal [28]. In order to balance performance and resource consumption, in this paper, the number of decomposition layers J is set to 5.

Finally, only the approximation coefficients are retained for signal reconstruction, which can effectively remove high-frequency noise. The signal reconstruction process is shown in Equation (9):

(9) $$x^{\prime }[t]=\sum_{k}cA\left[k\right]\cdot \emptyset_{J,k}\left(t\right)$$

where $x^{\prime }[t]$ represents the signal that has been reconstructed. This approach strengthens the main components of the signal and suppresses high-frequency noise in the level of detail.

**Figure 8:**
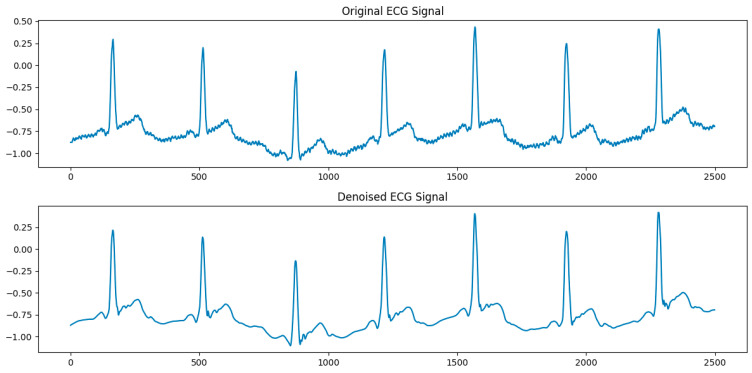
Comparison of ECG signals before and after DWT denoising.

The wavelet denoising results are shown in Figure 8.

It can be seen that the noise waveform has been significantly eliminated, while the linear correlation of the signal is quite strong. In order to provide a quantitative analysis of the denoising effect, three specific index parameters are introduced to be used for evaluation: the signal-to-noise ratio (SNR), root mean square error (RMSE), and correlation coefficient (CC). These parameters are defined in Equations (10)–(12):

(10) $$SNR=10\log_{10}\left(\frac{Signal\;Power}{Noise\;power}\right)$$

(11) $$RMSE=\sqrt{\frac{1}{n}\sum_{i=1}^{n}{\left(y_{i}-y_{i}^{\prime }\right)}^{2}}$$

where $y_{i}$ is the ith point of the original signal, $y_{i}^{\prime }$ is the ith point of the denoised signal, and n is the total number of data points in the signal. Here, RMSE focuses more on the quantization error of the signal (i.e., the magnitude of the difference between the predicted value and the actual value):

(12) $$CC=\frac{\sum \left(x-\bar{x})(y-\bar{y}\right)}{\sqrt{{\sum \left(x-\bar{x}\right)}^{2}{\sum \left(y-\bar{y}\right)}^{2}}}$$

where x and y represent the data points of the two signals and $\bar{x}$ and $\bar{y}$ are the mean values of x and y, respectively. Summing is carried out for all paired data points (x,y). CC provides a measure of the linear relationship between two signals, with values closer to 1 or −1 indicating a stronger linear relationship.

The results of the three evaluation indicators are shown in Table 4.

The outcomes reflected by these performance metrics demonstrate that the DWT denoising process is effective, significantly reducing noise while preserving the signal’s primary characteristics. Such improvement is instrumental in enhancing the accuracy of downstream analyses, including feature extraction and disease diagnosis.

(3) Comparison of Signal Processing Methods

To underscore the effectiveness of integrating a Kalman filter with a DWT for ECG signal denoising, this study also examined three established signal processing techniques—the Fourier transform, adaptive filter, and independent component analysis (ICA)—for a comparative performance analysis. The findings are presented in Table 5.

The results clearly demonstrate that the combination of the Kalman filter with the DWT, as utilized in this research, outperformed the others. Conversely, ICA was less effective, likely due to its prevalent application in multi-channel analyses.

(4) Correction for Baseline Drift

Baseline drift is a common problem in ECG signals, caused mainly by respiratory movements and small changes in electrode position. Methods of calibration usually include high-pass filtering and polynomial fitting and subtraction.

(1) High-pass filtering: A high-pass filter can be used to effectively remove baseline drift by setting a suitable cutoff frequency (usually less than 1 Hz). For example, a 0.05 Hz high-pass filter can be used to remove low-frequency interference caused by breathing.

For this research, a Butterworth filter was employed to establish a high-pass filter. This approach effectively removes baseline drift occurring at low frequencies while concurrently preserving the ECG signal components at higher frequencies. The high-pass filtering can be represented by the following transfer function in Equation (13), in which $f_{c}$ is the cutoff frequency:

(13) $$H\left(f\right)=\frac{f}{f+f_{c}}$$

where $f_{c}$ was set to 0.5 Hz, which was based on the value commonly used in ECG signal processing for effectively removing baseline drift without affecting the main frequency components of the ECG signal. Meanwhile, the order of the filter was set to 5 in order to balance the filtering effect and computational complexity.

(2) Polynomial fitting and subtraction: A more sophisticated approach is to fit a polynomial to the baseline of the ECG signal and then subtract this baseline model from the original signal. This method can dynamically adapt to baseline changes in the signal. However, this process involves inverse operations of matrices, and matrix multiplication requires a great amount of arithmetic power in its operation [29]. Moreover, its solution process is rather sensitive to small changes in the input data and requires additional numerical stability measures such as regularization to handle it. This method is difficult to apply to application scenarios with limited computing power or which require real-time processing. Therefore, it was not chosen in this study.

**Figure 9:**
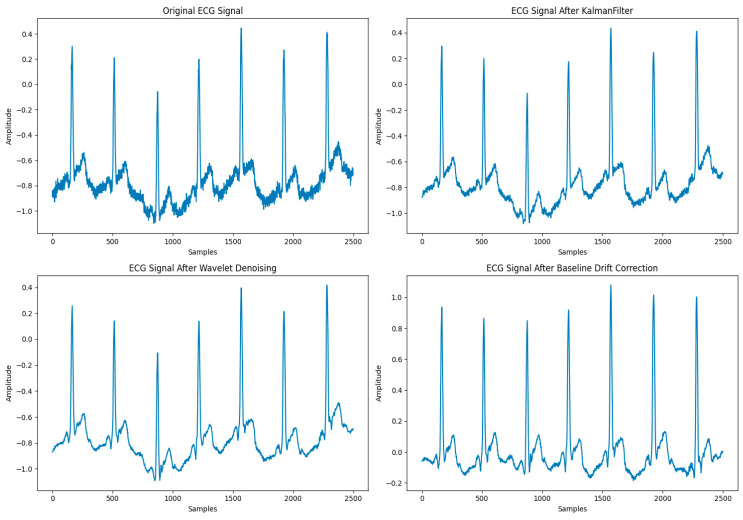
Comparison of ECG signals before and after denoising and baseline drift correction.

Figure 9 illustrates a comparison of the ECG signals before and after denoising and baseline drift correction. The signal-to-noise ratio improved by 37%, which greatly improved the signal quality.

This refinement not only decisively expunged noise and interference but also guaranteed the preservation of essential physiological information within the signal. This elevated signal quality lays a robust groundwork for ensuing signal analysis and feature extraction, securing the reliability of the processed data.

#### 2.4.2. EEG Signal Processing

By utilizing a DWT coupled with soft thresholding, the wavelet basis Ψ(t) and scale function φ(t) of the adapted processed signal were set, and the denoised EEG signal was taken out through signal reconstruction as shown in Equation (14):

(14) $$R_{a\times b}^{\prime }=DWT(R_{a\times b},\Psi (t),\varphi (t))$$

where the input of captured brainwave signals in $R_{a\times b}$. The signal is decomposed into a series of wavelet coefficients using the discrete wavelet transform (DWT). Unlike the continuous wavelet transform, which produces a large amount of redundant information when processing signals, the discrete wavelet transform is used to effectively remove the high-frequency noise in the original data and retain the low-frequency effective data by discretizing the translation factor and scale factor in the continuous wavelet transform [30].

As in the case of ECG signal processing, Daubechies wavelets with smoother shapes and longer support lengths are used as wavelet bases for EEG signal processing, with the number of layers set to 5 and the detail components set from level 1 to level 5, which roughly correspond to the bands β, α, θ, δ, and γ, respectively. In addition, a filter bank is generated by the wavelet and the scaling function for iterative filtering and downsampling of the signals as shown in Equations (15) and (16):

(15) $$A_{j}\left(t\right)=\sum_{k}h_{k}\cdot A_{j+1}(2t-k)$$

(16) $$D_{j}\left(t\right)=\sum_{k}g_{k}\cdot A_{j+1}(2t-k)$$

where $h_{k}$ and $g_{k}$ are the low-pass and high-pass filter coefficients of the wavelet, respectively, $A_{j+1}$ is an approximation factor for the upper level, and $D_{j}$ is the detail factor for the current level.

The threshold λ is determined based on the distribution of the wavelet coefficients, and a common method for this is to set the threshold to half of the standard deviation of the coefficients, which is calculated by the formula shown in Equation (17):

(17) $$\lambda =\frac{np.std(coeffs[-level])}{2}$$

where coeffs[−level] denotes the coefficient of the most detailed layer after wavelet decomposition and np.std is a function that calculates the standard deviation. This threshold will be used for soft thresholding for noise reduction or removal.

Then, soft thresholding is applied to all wavelet coefficients to attenuate or remove the noise component while maintaining as much signal detail as possible:

(18) $$\hat{c}=sign(c)\cdot max(\left|c\right|-\lambda ,0)$$

where c is the wavelet coefficients, λ is the calculated threshold, and $\hat{c}$ is the thresholded coefficients. This means that coefficients less than the threshold are set to 0, and coefficients greater than the threshold λ are subtracted. This processing removes the noise and maintains the shape of the signal, and it is particularly effective in maintaining important features of the signal.

**Figure 10:**
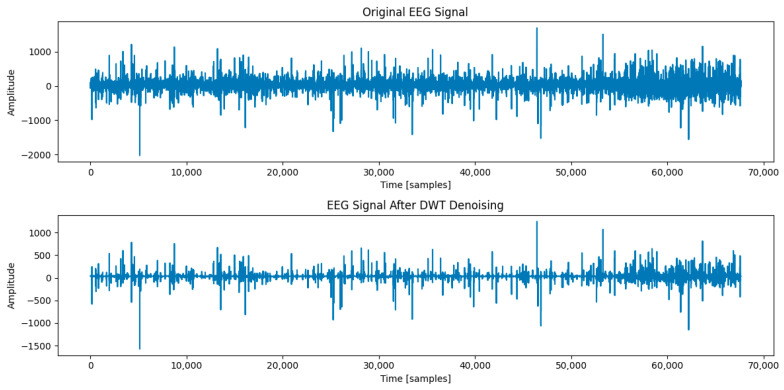
Comparison of EEG signals before and after DWT denoising.

The EEG results before and after denoising are shown in Figure 10.

The results of the three evaluation indicators regarding EEG signals after noise reduction by a DWT are shown in Table 6.

#### 2.4.3. Remove Industrial Frequency Noise

Finally, a Butterworth band-stop filter is utilized to remove the possible 50 Hz industrial frequency interference from the signal with Equation (19):

(19) $$H(f)=\frac{1}{\sqrt{1+{\left(\frac{f^{2}-f_{0}^{2}}{f\times BW}\right)}^{2n}}}$$

where $f_{0}$ is the center frequency to be removed (50 Hz), BW is the bandwidth (49–51 Hz), and n is the filter order, which is set to 4.

Finally, the EEG signal matrix and ECG signal vectors $R_{a\times b}^{\prime \prime }$ and $V_{ECG}^{\prime \prime \prime }$ after eliminating the IF can be obtained as shown in Equation (20):

(20) $$\left[R_{a\times b}^{\prime \prime },V_{ECG}^{\prime \prime \prime }]={filter}_{band}([R_{a\times b}^{\prime },V_{ECG}^{\prime \prime }],f_{0},BW,n\right)$$

### 2.5. Feature Extraction

#### 2.5.1. EEG Feature Extraction

The PSD extracted using Welch’s method demonstrated strong feature correlation in studies correlating EEG signals with fatigue, and it is also a good feature representation of the EEG signal [31]. The relationship between differential entropy (DE), cragginess (KS), skewness (SK), and fatigue is usually reflected in their close correlation with changes in the state of brain activity when used as physiological and neurobiological indicators.

(1) Power Spectral Density (PSD)

For an EEG signal $x\left(n\right),n=0,1,\ldots ,N-1$ of a length N, which is split into L ends, the PSD of the signal $x_{l}(n)$ is defined as shown in Equation (21):

(21) $$P_{l}\left(f\right)=\frac{1}{MU}{\left|\sum_{n=0}^{M-1}x_{l}(n)\omega (n)e^{j2\pi fn}\right|}^{2}$$

where l=0,1,…,L−1, M is the length of each signal segment, ω(n) is the additive window function (Hamming window), and the regularization coefficient U of the window is

(22) $$U=\frac{1}{M}\sum_{0}^{M-1}\omega^{2}(n)$$

The power spectral density of the signal $x\left(n\right)$ is obtained by averaging the power spectral densities of all the L-band signals, which is calculated as follows:

(23) $$P\left(f\right)=\frac{1}{L}\sum_{l=0}^{l-1}P_{l(f)}$$

(2) Differential Entropy (DE)

High DE may indicate increased uncertainty in brain activity, associated with high cognitive activity or mental load, and is the most accurate and stable EEG feature reflecting changes in human alertness [32]:

(24) $$DE=\frac{1}{2}{log}_{2}(2\pi e\cdot var(EEG))$$

(25) $$var\left(X\right)=\frac{1}{N}\sum_{i=1}^{N}{\left(x_{i}-\mu \right)}^{2}$$

where $x_{i}$ is the $i_{th}$ value in the signal, μ is the average of all values in the signal, and N is the total number of values in the signal. The greater the variance, the more erratic the variation in the signal. In the context of DE, variance is used to indicate the uncertainty of a signal, and DE is used to quantify the complexity or information content of a signal.

(3) Kurtosis (KS)

In fatigue monitoring, changes in KS may indicate changes in the efficiency of cognitive processes or adjustments in the way the brain processes information [33]:

(26) $$KS=\frac{E[{\left(X-\mu \right)}^{4}]}{\sigma^{4}}$$

where E denotes the expected value, X is a random variable, μ is the mean of X, and σ is the standard deviation of X. The equation measures the sharpness of the shape of the probability distribution, reflecting how sharp or flat the data distribution is relative to the normal distribution.

(4) Skewness (SK)

Under certain mental states (e.g., when fatigued or highly focused), the skewness of the brain’s electrical activity may change, thus reflecting changes in the way the brain processes information or responds to mental load [34]:

(27) $$SK=\frac{E[{\left(X-\mu \right)}^{3}]}{\sigma^{3}}$$

where E denotes the expected value, X is a random variable, μ is the mean of X, and σ is the standard deviation of X. This formula is used to measure the degree of asymmetry in the distribution of data, with positive values indicating that the distribution is right-skewed, negative values indicating that the distribution is left-skewed, and a value of 0 indicating that the distribution is symmetric.

#### 2.5.2. ECG Feature Extraction

Analysis of ECG signals typically centers on heart rate variability (HRV), a key indicator of cardiac and autonomic nervous system functions. In this experiment, representative features of HRV in the time and frequency domains were selected.

(1) Heart Rate

Heart rate (HR) is one of the important features in ECG analysis. It is calculated as shown in Equation (28):

(28) $$HR=\frac{\sum_{i=1}^{I}\frac{60}{RR_{i}}}{I}$$

where I is the total number of R-R intervals within the current signal.

(2) Root Mean Square of Successive Difference

The RMSSD represents the root mean square of the difference between all neighboring R-R intervals over time. The calculation process is shown in Equation (29):

(29) $$RMSSD=\sqrt{\frac{1}{HR-1}\sum_{i=1}^{HR-1}{\left(RR_{i+1}-RR_{i}\right)}^{2}}$$

where $RR_{i+1}$ and $RR_{i}$ denote the ${i+1}_{th}$ and $i_{th}$ R-R intervals, respectively. The RMSSD reflects the variation of adjacent cardiac beat cycles.

(3) Frequency Domain Indicator

Studies have demonstrated that the LF, HF, and LF/HF are highly correlated with fatigue, and the spectral range of HRV is generally 0–0.5 Hz. In clinical practice, HRV is usually divided into low-frequency (LF) and high-frequency (HF) components, using 0.15 Hz as a cut-off point:

(1) LF: 0.04 Hz–0.15 Hz, reflecting cardiac sympathetic nerve activity;

(2) HF: 0.15 Hz–0.4 Hz, reflecting the activity of the cardiac vagus nerve;

(3) LF/HF: the ratio of low-frequency power to high-frequency power, reflecting the stability of sympathetic and vagal activity.

When analyzing the LF and HF components over short durations, their respective data can be significantly impacted by the TP, leading to potential distortions in their absolute values. To mitigate this, it is advisable not to rely on direct comparisons of absolute values. Instead, normalization against the very low-frequency (VLF) band, which is below 0.04 Hz, is employed to adjust LF and HF values. This normalization process ensures a more accurate representation by minimizing the influence of the total power on the LF and HF bands as shown in Equations (30)–(32):

(30) $${LF}_{norm}=\frac{LF}{TP-VLF}$$

(31) $${HF}_{norm}=\frac{HF}{TP-VLF}$$

(32) $$\frac{LF}{HF}=\frac{{LF}_{norm}}{{HF}_{norm}}$$

The ECG feature row vector consists of HR, RMSSD, ${LF}_{norm}$, ${HF}_{norm}$, and LF/HF indicators in that order.

#### 2.5.3. Feature Preference

Maximum relevance minimum redundancy (mRMR) is a filtered feature selection algorithm that uses mutual information as a criterion for judging feature-to-feature and feature-to-category relevance, and it ultimately calculates a score for each feature. The expression of the mutual information is shown in Equation (33):

(33) $$I(x;y)=\iint p(x,y)log\frac{p(x,y)}{p(x)p(y)}dxdy$$

where I(x;y) is the mutual information of the feature parameters x,y, and $p\left(x\right),\;p(y)$, and p(x,y) are the respective probability densities and joint probability density.

To find the subset of features Q containing individual features, the maximum relevance principle searches for the best n features $x_{i}$ related to the target category c in the proper order of $I(x_{i};c)$. Computation is performed as shown in Equation (34):

(34) $$maxD(Q,c);D=\frac{1}{\left|Q\right|}\sum_{x_{i}\in Q}I(x_{i};c)$$

Then, the maximum correlation coefficient and minimum redundancy were integrated using the operator. The calculation process is shown in Equation (35):

(35) $$max\Phi \left(Q,c\right);\Phi =D-R$$

where the merit ranking of each feature quantity in the sample set Q can be calculated.

**Figure 11:**
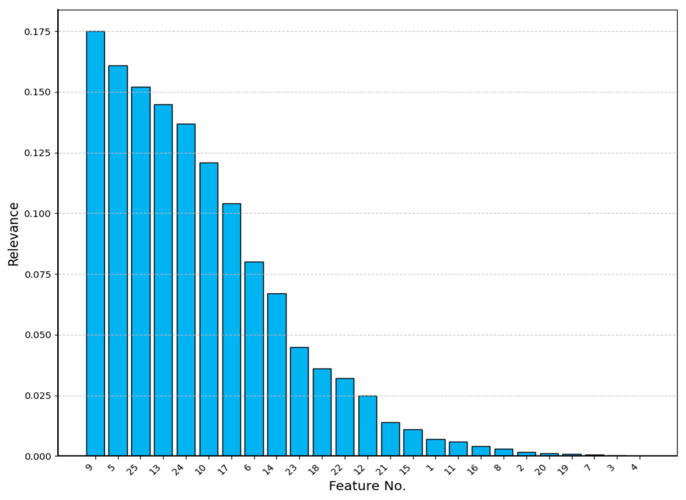
Feature relevance ranking results.

All of the features were numbered and sorted. The EEG was sorted from δ to γ for the PSD, DE, KS, and SK, corresponding to numbers 1–20, and the ECG was numbered from 21 to 25 for the HR, RMSSD, ${LF}_{norm}$, ${HF}_{norm}$, and LF/HF. Assuming that the number of the best feature set was 25, the results of the superiority ranking of all feature quantities are shown in Figure 11.

**Figure 12:**
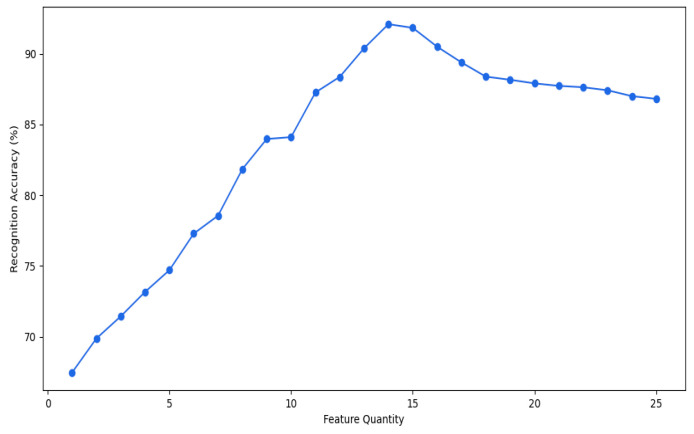
Classification accuracy under different feature sets.

Ultimately, the process iteratively increased the number of features in the optimal set, starting from one. For each iteration, the gcForest model was trained and tested using the default parameter configurations. This iterative approach enabled the identification of the optimal feature set size that yielded the best recognition accuracy. The resulting accuracies for each feature set size are presented in Figure 12, illustrating the model’s performance across different configurations.

The analysis indicates that utilizing the top 14 ranked features to create a feature subset culminated in the highest recognition accuracy, peaking at 92.10%. Beyond this optimal feature set size, the accuracy began to marginally decline. Consequently, this study proceeded to leverage these top 14 features identified through the screening process for the analysis of fatigue state recognition, demonstrating their efficacy in achieving the most accurate model performance.

#### 2.5.4. Multi-Modal Feature Fusion

**Figure 13:**
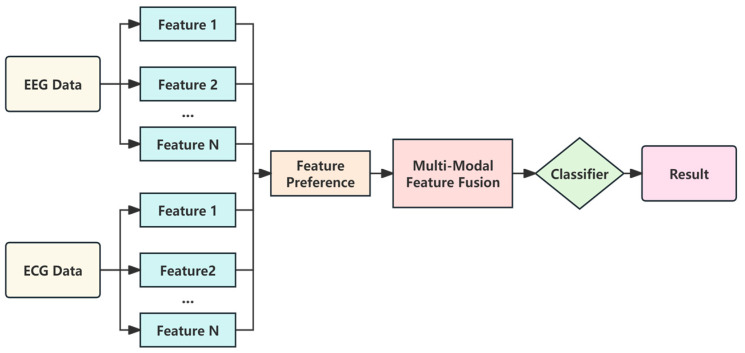
Multi-modal feature fusion framework.

The multimodal feature fusion dataset was constructed by fusing the data acquired from different sensors through feature extraction and then fusing them into a larger feature vector through the feature cascade process. The aim was to improve the prediction performance of the machine learning model by utilizing the complementary information of the different modalities, and the framework is shown in Figure 13.

For a dataset of a time length t, the process of computing the feature fusion matrix using the sliding window method is as follows.

A suitable window size W (30 s) and step size S (1 s) are selected, and an empty feature matrix F is initialized for storing the feature vectors of all time windows. The time range for each window is shown in Equation (36):

(36) $$W=[t_{i},t_{i}+W],t_{i}=t_{0}+i\times S$$

Then, from the start time $t_{0}$ to the end time t, the window is moved according to the step size S, and the 9 features contained in the EEG and the 5 features contained in the ECG are extracted within each window to form a 14 dimensional feature vector, for the ith window the feature vector $f_{i}$, which can be expressed as shown in Equation (37):

(37) $$f_{i}=[f_{EEG1},f_{EEG2},\ldots ,f_{EEG9},f_{ECG1},f_{ECG2},\ldots f_{ECG5}]$$

where $f_{EEGx}$ and $f_{ECGy}$ are the results of feature computation for the EEG and ECG, respectively.

After successively recording the feature vectors of each window, the whole feature matrix F is stacked by the feature vectors of all windows in chronological order as shown in Equation (38):

(38) $$F=\left[\begin{array}{c} f_{1}\\ f_{2}\\ \begin{array}{c} \vdots \\ f_{i} \end{array} \end{array}\right]$$

The final feature fusion matrix F is used as input to the recognition model for subsequent data analysis and model learning training.

### 2.6. Brain Fatigue State Recognition Model Based on Bayes-gcForest Algorithm

**Figure 14:**
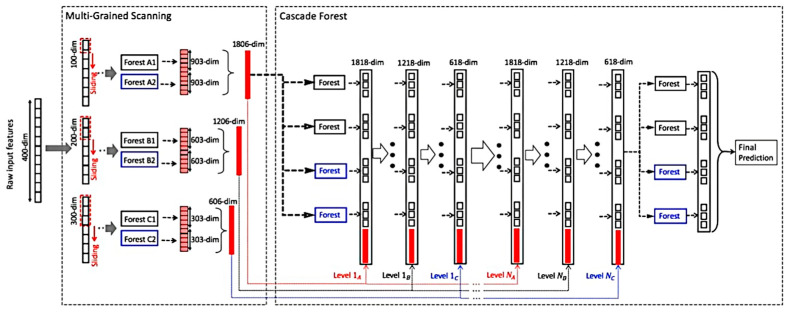
Structure of the gcForest model.

Since its debut in 2019, the gcForest model has garnered significant interest across various domains, showcasing its potential for both scholarly research and practical applications. The essence of the model resides in its innovative architecture: a deep forest structure that integrates multiple cascaded random forests. This design philosophy is pivotal to the model’s success and is illustrated in Figure 14, highlighting its foundational approach to leveraging ensemble learning for improved predictive performance.

For each level of cascade forest, its output depends not only on the current input data but also on the output of the previous level of the cascade forest, and each level of the cascade forest can be represented as shown in Equation (39):

(39) $$F_{i}(X)=RF_{i}(X⊕F_{i-1}(X)$$

where X is the input feature set, $RF_{i}$ denotes the random forest model at layer i, and ⊕ represents the feature linking operation.

In the cascade forest structure, except for the first layer, whose input is the original feature vector $X_{0}$, each layer is the combination of the original feature vector with the augmented feature vector $X_{i}$ generated by the previous layer. This combination allows the information from the original feature space to always be preserved and applied in every process. As the layers continue to be stacked, the valid information in the features is continuously enhanced. When the final layer is reached, the output feature vectors will no longer be combined with the original feature vectors but will participate in the classification as the final class vector $X_{last}$ as shown in Equation (40):

(40) $$X_{last}=X_{0}+\sum_{i=1}^{i}X_{i}$$

The final class vector $X_{last}$ contains the probability of classifying the current sample, where the class with the highest probability $MAX\left(X_{last}\right)$ is the cascade forest classifier’s estimate R of whether brain fatigue is currently present:

(41) $$R=MAX\left(X_{last}\right)$$

#### Hyperparameter Optimization Process of Bayes-gcForest

**Figure 15:**
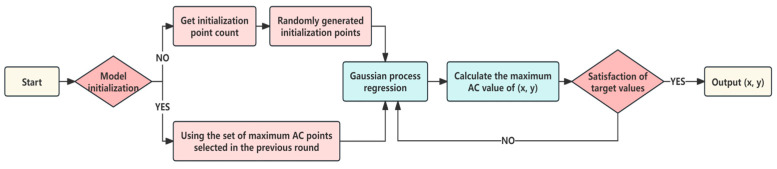
Bayesian optimization flowchart.

The hyperparameters of the gcForest model align closely with those typical of deep forest configurations, particularly emphasizing the significance of parameters such as the number of random forests per layer (n_cascadeRF) and the number of trees within each random forest in the cascade (n_cascadeRFtree). To optimize these critical parameters efficiently, a Bayesian optimization approach was employed, a process specifically detailed in Figure 15. This methodological choice for hyperparameter tuning underpins the model’s adaptability and performance optimization, showcasing a structured approach to enhancing the gcForest model’s efficacy.

(1) Firstly, the objective function $f\;(n_{cascadeRF},n_{cascadeRFtree})$ is modeled using a Gaussian process (GP):

(42) $$f\sim GP(m(x),k(x,x^{\prime })$$

where m(x) is the mean function and $k(x,x^{\prime })$ is the kernel function used to define the similarity of any two points in the parameter space.

Then, the set initial parameter point $x\;(n_{cascadeRF\_initial},n_{cascadeRFtree\_initial})$ is selected for evaluating the performance of the current gcForest model, and the initial dataset D is constructed and utilized to update the GP model, setting the number of iterations i to 50.

(2) Secondly, during each update iteration, Bayesian optimization selects the next parameter point $x_{next}$ by solving the following optimization problem:

(43) $$x_{next}={argmax}_{x}EI(x;D_{current})$$

(44) $$D=D\cup \left\{{(x}_{next},f(x_{next}))\right.$$

where EI(x) is the expected improvement (EI) of the acquisition function and $D_{current}$ is the current set of observations.

(3) Then, k-fold cross-validation is given to the optimization process, which is used to accurately assess the performance of the model under each set of hyperparameter configurations. The process definition is shown in Equation (45):

(45) $$f(x)=\frac{1}{k}\sum_{i=1}^{k}P_{i}(x)$$

where k is the number of folds, which is set to five in this paper, and $P_{i}(x)$ represents the performance index of model f(x) at the $i_{th}$ test.

(4) Finally, after continuous update iterations, the optimization process is terminated when the upper limit of the number of iterations is reached or the model no longer shows significant performance improvement:

(46) $$EI(x)=E\left[max(0.f(x)-f(x^{+}))\right]$$

where EI(x) is the expected improvement and $f(x^{+})$ is the best objective function value observed up to the current number of iterations. After obtaining the optimal parameters $n_{cascadeRF\_best}$ and $n_{cascadeRFtree\_best}$, they are used for subsequent predictions of the model.

## 3. Results and Discussion

### 3.1. Bayesian Optimization Experiments

To validate the performance enhancements of the Bayes-gcForest model introduced in this study, the research incorporated three optimization techniques—grid search, random search, and Bayesian optimization—each applied to the foundational gcForest model. The effectiveness of the optimal hyperparameters identified by these methods was assessed based on their influence on the classification accuracy. This assessment utilized the accuracy (Acc) metric to quantify the performance across various tuning and optimization scenarios. The calculation of the classification accuracy is formalized in Equation (47), providing a quantitative basis for comparing the impact of each optimization strategy on the model’s predictive accuracy:

(47) $$Acc=\frac{TP+TN}{TP+TN+FP+FN}$$

where TP, TN, FP, and FN stand for true positive cases, true negative cases, false positive cases, and false negative cases, respectively.

This study utilized data from the subject labeled “Test1” to assess the impact of the number of trees within the model on the classification accuracy, where $n_{cascadeRF}$ was pre-set to take the value range of (1–10) and $n_{cascadeRFtree}$ took the value range of (10–300), and the results of the optimization search are shown in Table 7.

**Figure 16:**
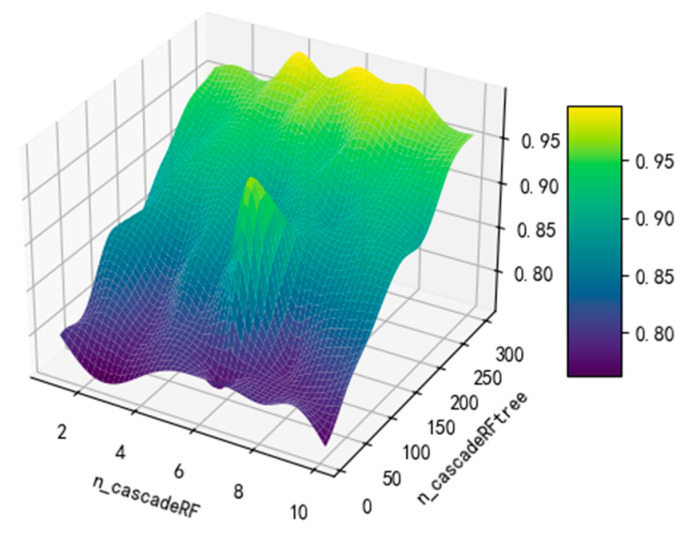
Impact of hyperparameters on model performance.

The outcomes reveal that, given the straightforward characteristics of the dataset employed in this experiment, both Bayesian optimization and grid search attained an optimal recognition rate of 97.2%. However, the grid search method, which exhaustively iterates through all possible hyperparameter combinations to identify the best solution, significantly increased the total computational time due to its exhaustive nature. This observation highlights a trade-off between the accuracy of the model and the efficiency of the hyperparameter optimization process. The effect of hyperparameters on model performance during Bayesian optimization is shown in Figure 16.

### 3.2. Formal Verification

With escalating computational load and system complexity, there is a corresponding increase in the demand for model stability and robustness [35]. Therefore, this study uses an approach based on abstract interpretation to analyze the model behavior and help the researcher to better understand the decision-making process of the model and verify that it conforms to the expected behavior.

(1) Model Validation

Test cases were established for the critical performance metrics, including model accuracy, recall, precision and F1 score, supplemented by corresponding unit tests. In this research, thresholds for all four indicators were established at a minimum of 0.90.

After testing, the model was able to meet the performance requirements for all the data classification results.

(2) Model Checking

**Figure 17:**
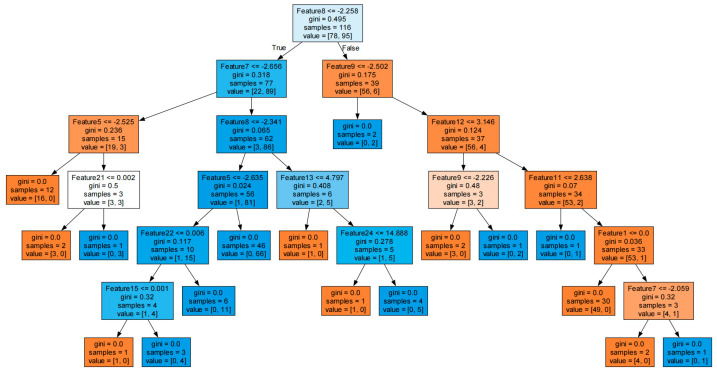
Visual decision tree.

Incorporating the decision boundaries of the visual decision tree within the model’s code facilitates an understanding of the model’s decision making within specific feature spaces [36]. The results from these tests are depicted in Figure 17.

Figure 17 shows how the model recursively splits the data based on different features and thresholds. Eventually, the data are split into leaf nodes, and the model makes predictions based on the majority category of the samples that arrive at each leaf node.

Here, “Feature < X” instructs the model to segment the data using that feature value at that node, “gini” represents the Gini impurity of the node, which is a value ranging from 0 to 1, with 0 indicating that all samples belong to the same category (the highest purity) and values close to 1 indicating that the samples are evenly distributed across the categories (the lowest purity), “samples” denotes the number of samples arriving at the node, and “value” denotes the number of samples in that node for each category.

The Gini impurity of each leaf node was 0, which means that the samples in the node all belonged to the same category, and the model categorized them effectively.

(3) Adversarial Testing

Adversarial testing is a method of evaluating the robustness of a model to small, intentional input perturbations. In machine learning, this usually means modifying the test data in an attempt to “trick” the model and cause false predictions. The first step is to add some random noise to the test data. The key to this step is to add noise that is not so large that it completely alters the true class of the data but also small enough to test the sensitivity of the model to small disturbances.

The results of model training with the inclusion of adversarial tests of different intensities are shown in Table 8.

The findings indicate that low-intensity random noise introduced minimal perturbations to the model. Even with noise levels approaching 0.5, the model sustained a recall rate exceeding 91.31%. At a noise level of one, representing extreme noise conditions akin to adversarial testing, the model nearly obscured the original data signal yet retained a recall of 81.02%, evidencing its robustness.

### 3.3. Experiments on Fatigue State Recognition Based on Bayes-gcForest

**Figure 18:**
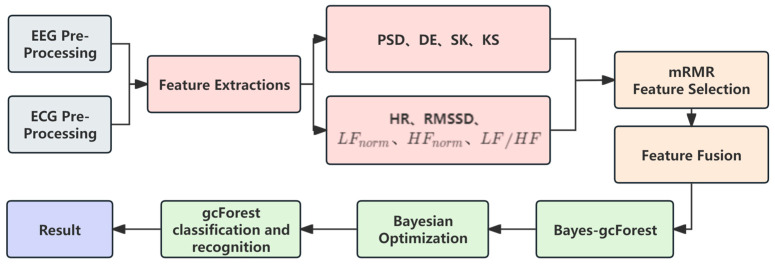
Brain fatigue recognition flowchart.

Figure 18 presents the workflow of brain fatigue recognition utilizing the Bayes-gcForest approach. The process begins with the preprocessing of raw data, which encompasses labeling and segmenting the data into different frequency bands. Subsequently, feature extraction is carried out on both EEG and ECG data, yielding a total of 9 classes and 25 distinctive features. These features are then optimized using the mRMR algorithm, forming a multimodal feature fusion matrix. This optimized set of features is finally inputted into the fatigue recognition model for classification.

#### 3.3.1. Model Accuracy Evaluation

**Figure 19:**
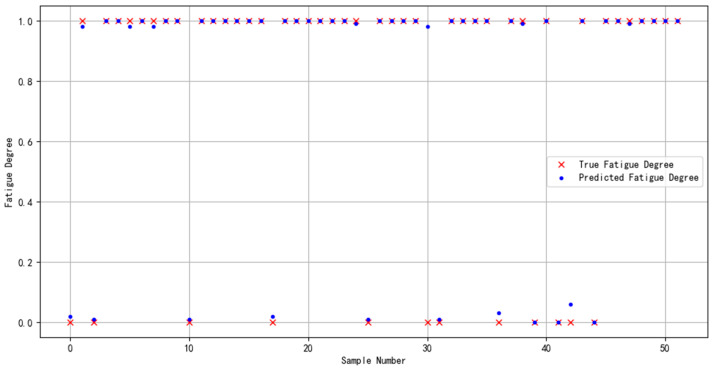
Predictive results of multimodal feature fusion for brain fatigue state recognition.

The dataset specifically constructed for this study was partitioned following a ratio of “60% for training, 20% for validation, and 20% for prediction”. This distribution guaranteed an adequate volume of data for model training while simultaneously ensuring that the validation and prediction phases were supported by sufficient sample sizes. Such a structured approach facilitated efficient model selection and enabled a thorough evaluation of the model’s generalization capability. The outcomes of the prediction phase are depicted in Figure 19.

The data would be targeted for the effective evaluation of the model binary classification effect, and the results are shown in Table 9.

In order to demonstrate the ability of multimodal fusion features to improve the classification performance, this study also compares the classification results of the multimodal fusion features (EEG and ECG) and unimodal features (EEG or ECG), as shown in Table 10. The optimal average recognition rate of 96.13% was obtained for the multimodal fusion features, while the average recognition rates of 93.26% and 88.78% were obtained for the EEG features and ECG features alone, respectively. The analysis of the constructed brain fatigue dataset revealed that the classification performance using a single modality, whether it be EEG or ECG features, was already commendable. However, the integration of multiple modalities within the feature set markedly enhanced the classification accuracy. This improvement underscores the value of combining various data sources, demonstrating that the synergy of multimodal features significantly amplifies the model’s ability to discern fatigue states with greater precision.

The observed standard deviation in the classification outcomes for the multimodal fusion feature was notably low at 1.47%, considerably less than that associated with unimodal features. This highlights the enhanced stability and robustness afforded by the incorporation of multimodal features into the model, demonstrating its superior adaptability across diverse subject data.

**Figure 20:**
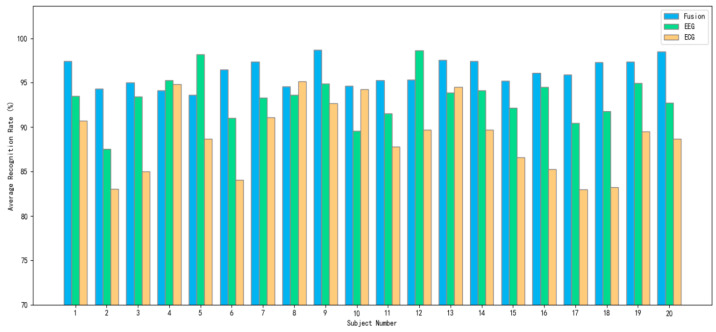
Comparison of unimodal and multimodal recognition prediction results.

To obtain a better visualization of the impact of fused and unimodal features on each subject’s data, Figure 20 shows the average recognition rate obtained by the model for 20 subjects’ data with different modal features.

The analysis revealed that over 80% of the subjects exhibited a higher average recognition rate with multimodal fusion features compared with unimodal features. Furthermore, the variation of multimodal fusion features in the overall recognition rate was minimized, indicating enhanced model stability. This suggests that multimodal fusion features not only bolster the overall classification performance across varied subject data but also lead to more consistent and accurate outcomes. Essentially, the integration of multiple data modalities effectively harnesses the strengths of each, resulting in a robust model capable of adapting to and accurately classifying diverse types of data from different subjects.

#### 3.3.2. Model Validation Experiment

**Figure 21:**
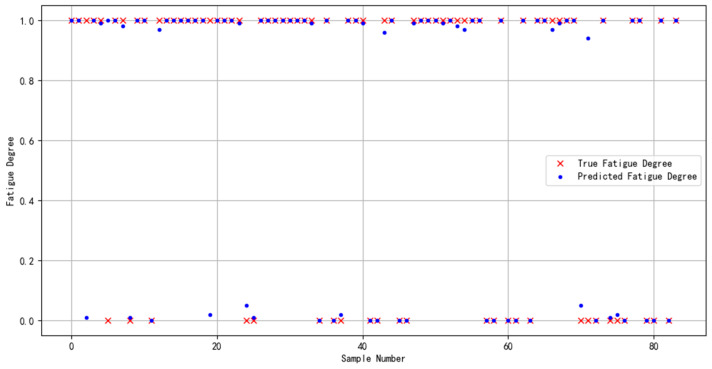
DROZY dataset’s identified predicted results.

In order to demonstrate the effectiveness and compatibility of the Bayes-gcForest algorithm model proposed in this paper for brain fatigue recognition when oriented to different datasets, the multi-featured fatigue dataset (DROZY) [37] released in 2016 by the Laboratory for Signal and Image Exploitation (INTELSIG), which is part of the Department of Electrical Engineering and Computer Science of the University of Liège (ULg) in Liège, Belgium, was used as a validation experimental dataset for the experiments, and the prediction results are shown in Figure 21.

From the model evaluation indexes, the recall value was 0.9571, the precision value was 0.9501, and the F1 score was 0.9534. From the results, it can be seen that the model’s prediction effect for this dataset was effective. Compared with the previous fatigue detection study for the same dataset, which only used the method of extracting ECG or EEG features, the method used in this study improved the results by 5.74% [38] and 5.71% [39], respectively. The above results fully validate the compatibility and effectiveness of the Bayes-gcForest algorithmic model proposed in this paper for the identification and valuation of datasets from different sources, especially small-scale datasets.

#### 3.3.3. Model Comparison Experiment

**Figure 22:**
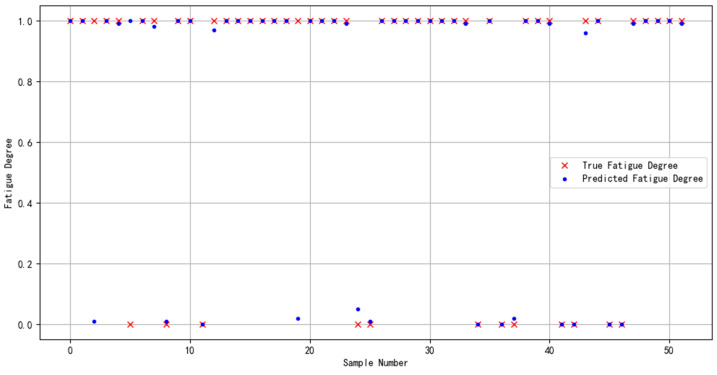
gcForest prediction diagram.

**Figure 23:**
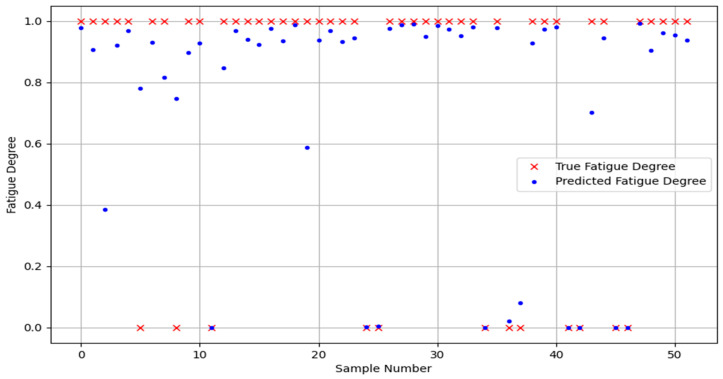
SVM prediction diagram.

**Figure 24:**
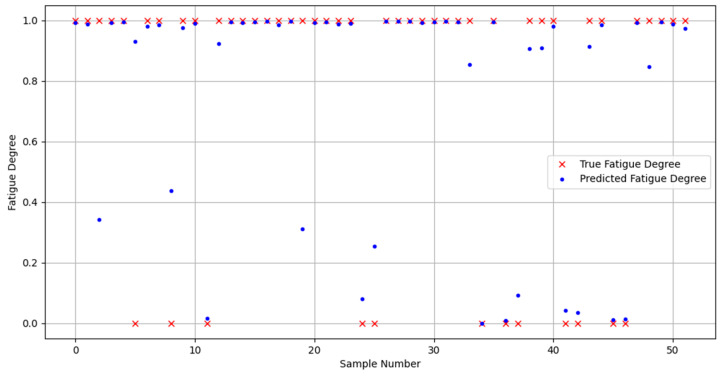
BP prediction diagram.

**Figure 25:**
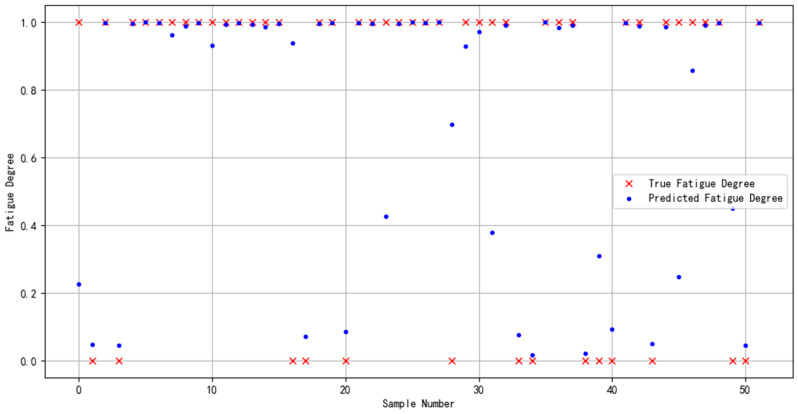
CNN prediction diagram.

**Figure 26:**
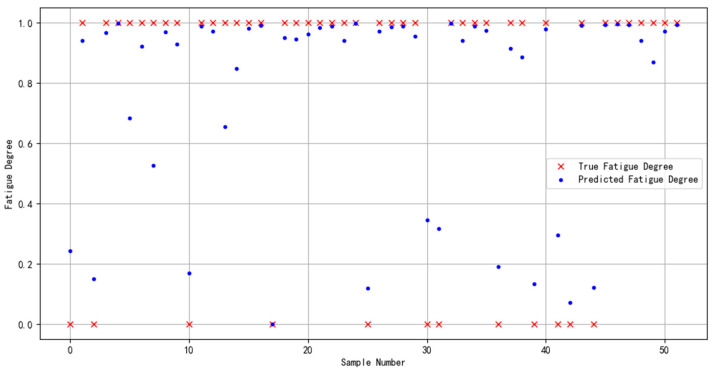
LSTM prediction diagram.

In order to prove the classification ability of the Bayes-gcForest algorithm model proposed in this paper, a total of five models, including unimproved gcForest, the SVM and BP machine learning methods, and the CNN and LSTM deep learning methods, were selected for performance comparison in the comparison experiments. The experiment results are shown in Figure 22, Figure 23, Figure 24, Figure 25 and Figure 26.

It can be seen that Bayes-gcForest achieved the highest average recognition rate of 96.13%, which was a significant advantage over all five other types of models. The unimproved gcForest model also achieved better performance than the other models. The deep learning model predictions generally performed better than machine learning. The SVM had the lowest average recognition rate of 77.51%. The classification performances of all models are shown in Table 11.

When analyzed from the perspective of the standard deviation, the stability performance of the Bayes-gcForest model was notably superior, enhancing its ability to accurately discern the fatigue states of subjects. This empirically demonstrates the efficacy of utilizing a fusion of features as model input for fatigue recognition, effectively compensating for the limitation where EEG variations are less pronounced at lower levels of fatigue.

**Figure 27:**
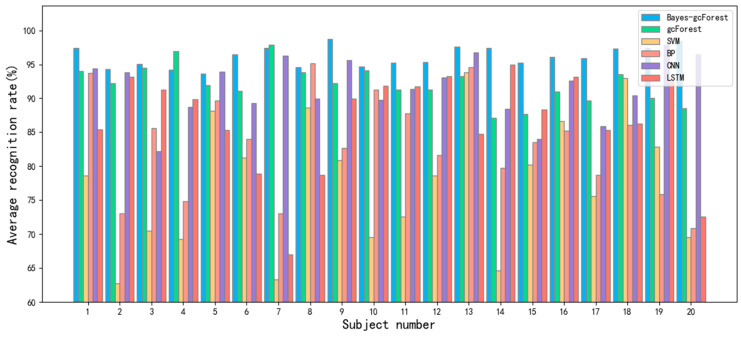
Comparison of recognition rates predicted by different models.

To obtain a better visualization of the impact of different models on each subject’s data, Figure 27 shows the average recognition rates obtained by the models using different modal features for the 20 subjects’ data.

Consequently, it can be concluded that the brain fatigue state recognition model based on Bayes-gcForest is more ideal for fatigue state discrimination and can be deeply applied to the subsequent fatigue state recognition for researchers.

## 4. Conclusions

This study designed and developed a portable, cost-effective, and user-friendly system with real-time capabilities for acquiring, monitoring, and analyzing physiological signals. Utilizing the enhanced Bayes-gcForest algorithm, the proposed method for brain fatigue state recognition attained a high average recognition rate of 96.13% on a bespoke dataset. Meanwhile, on the publicly available dataset DROZY, the model also achieved a recognition rate of 95.71%, which fully demonstrates the excellent prediction effect of the model on this dataset. Compared with the previous fatigue detection studies on the same dataset using only EEG or the fusion of EEG and ECG features, it improved the results by 5.74% and 5.71%, respectively. The brain fatigue recognition model proposed in this paper has high recognition accuracy and compatibility.

In summary, the system proposed in this study provides a solution to the problems of traditional EEG and ECG acquisition devices being expensive and difficult to operate and having poor portability. After passing relevant industry standard tests, the system can be widely applied to work scenarios that require focused monitoring of employee fatigue. By tracking and measuring the fatigue states of the staff to grasp their patterns, it can guide the relevant departments or enterprises to scientifically formulate a rest plan to improve work efficiency and safety.

### 4.1. Limitations and Directions for Improvement

However, due to the limitations of the experimental period, manpower, and related resource packages, this study still has some limitations:

(1) The anti-interference ability of the system needs to be further enhanced, which is important for promoting the application scope of the system.

(2) While this paper’s binary classification model reliably detected the presence of fatigue, fatigue itself is a multifaceted human factor issue that is challenging to categorize according to specific criteria [40]. Therefore, it is worth exploring how to better define and refine the classification criteria for fatigue.

(3) Due to the limitations of the experimental conditions, this study mainly focused on fatigue recognition through integrating EEG and ECG features. The introduction of more fatigue-related features can be considered in future work based on other research results.

(4) In this study, only 22–25 year-old regular and healthy graduate students in universities were selected as the subjects. This may be different from the actual physiological and psychological conditions as well as fatigue perception of research workers who have been in high-pressure research environments for a long time, and the diversity of the sample was more limited. Therefore, in follow-up research on brain fatigue recognition, the sample data should be increased by expanding the population and the range of characteristics of the research subjects, and the fatigue characteristics of research workers in specific industries should be further investigated under the premise of ensuring the diversity of the samples.

(5) The fatigue recognition model still has a greater demand for arithmetic resources, and it is temporarily unable to form an integrated portable mobile device with the collection system that has a limited arithmetic capacity.

### 4.2. Future Plan

Therefore, the future research direction mainly focuses on the following five points:

(1) Further improve the accuracy and anti-interference ability of sensors and hardware circuits;

(2) Increase the specific research on different fatigue levels and achieve more accurate identification of multi-classification problems through further optimization of the algorithm model;

(3) Add multiple physiological signals and features, such as EMG, EOG, and other physiological signals, to further enhance the accuracy and robustness of fatigue recognition by integrating these diverse fatigue indicators;

(4) Consider conducting further validation studies with larger sample sizes to strengthen the generalizability of the results;

(5) Improve the algorithm model to reduce the arithmetic resource demand while improving the model performance.

## Figures and Tables

**Table 1:** Experimental configuration.

Name	Parameters
OS	Windows 11 Professional Workstation Edition
Python	3.9.18
CPU	12th Gen Intel(R) Core (TM) i9-12900H 2.50 GHz
GPU	NVIDIA GeForce RTX 3080 Ti Laptop 16G (GDDR6)
RAM	Micron DDR5-4800 16 GB*2

**Table 2:** Criteria for subject selection.

Items	Specific Requirements
Health Condition	No history of cardiovascular, cerebrovascular, or mental illness and in good health
Exogenous Interferences Eliminated	Have not been exposed to any caffeine, nicotine, or other beverages, foods, tobacco, alcohol, or drugs that act or may act on the nervous system for at least one week prior to participating in the experiment
Daily Routine	Maintained a regular schedule with at least 8 h of sleep per day for one week prior to the start of the experiment
Other	Right-handed

**Table 3:** Classification of brain fatigue states.

Self-Scoring	Determination of Fatigue Status
1–5	No (N)
6–9	Yes (Y)

**Table 4:** Evaluation results of quantitative metrics of ECG denoised by DWT.

Evaluation Indicators	Results
RMSE	0.0195
SNR	32.1356
CC	0.9963

**Table 5:** Comparison of signal processing methods.

Signal Processing Methods	RMSE	SNR	CC
Kalman filter + DWT	0.0195	32.1356	0.9963
Fourier transform	0.7613	0.3054	0.9177
Adaptive filter (LMS)	0.0825	19.6076	0.9344
ICA	0.2405	9.5444	0.4010

**Table 6:** Evaluation results of quantitative metrics of EEG denoised by DWT.

Evaluation Indicators	Results
RMSE	0.0152
SNR	44.0618
CC	0.9999

**Table 7:** Comparison of hyperparameter optimization results.

Algorithms	Default or Optimal Parameters		Model Computing Time (s)	Accuracy (%)
	$n_{cascadeRF}$	$n_{cascadeRFtree}$		
Manual	2	101	10.41	92.1
Grid Search	3	250	831.78	97.2
Random Search	3	220	84.34	93.4
Bayesian Optimization	3	250	104.09	97.2

**Table 8:** Adversarial test results.

Random Noise Values	Average Recall before Test (%)	Average Recall after Test (%)
0.01	95.16	95.16
0.05	95.16	95.16
0.1	95.16	95.16
0.3	95.16%	91.93
0.5	95.16%	91.31
0.7	95.16%	81.64
1	95.16%	81.02

**Table 9:** Bayes-gcForest model classification recognition results.

Fatigue Category	Precision (%)	Recall (%)	F1 Score (%)	Standard Deviation (%)
No (N)	96.93	96.37	96.35	1.43
Yes (Y)	94.31	95.89	95.33	1.51
Average	95.62	96.13	95.84	1.47

**Table 10:** Comparison of classification results between unimodal and multimodal fusion features.

Type of Feature	Average Recognition Rate (%)	Standard Deviation (%)
EEG	93.26	2.63
ECG	88.87	4.10
EEG and ECG	96.13	1.47

**Table 11:** Comparison of classification performances of different models.

Model	Average Precision (%)	Average Recall (%)	Average F1 Score (%)	Standard Deviation
Bayes-gcForest	95.62	96.13	95.76	1.47
gcForest	95.01	92.10	93.53	2.67
SVM	81.36	77.51	79.39	9.36
BP	82.18	83.36	82.77	7.23
CNN	90.84	91.54	91.18	4.23
LSTM	89.96	86.72	88.02	7.24

## Data Availability

The “ULg Multimodality Drowsiness Database” (DROZY) data used in this study can be obtained from the following website: http://www.drozy.ulg.ac.be/, accessed on 11 October 2023.
